# Trajectories and influencing factors of social anxiety in postoperative breast cancer patients

**DOI:** 10.1186/s12888-024-05770-8

**Published:** 2024-05-14

**Authors:** Shaotong Wang, Yafang Hua, Yueyue Zhang, Daoxia Guo, Li Tian

**Affiliations:** 1 The First Affiliated Hospital of Soochow University, Suzhou, 215000 China; 2 School of Nursing, Medical College of Soochow University, Suzhou, 215000 China; 3 The First People’s Hospital of Kunshan, Suzhou, 215000 China; 4 The First Affiliated Hospital of Soochow University/ School of Nursing, Medical College of Soochow University, No. 188 Shizi Road, Suzhou, 215006 China

**Keywords:** Rumination, Social anxiety, Breast cancer, Trajectory changes, Growth mixture model

## Abstract

**Background:**

Social anxiety among postoperative breast cancer patients is a prevalent concern, with its intensity fluctuating throughout the course of treatment. The study aims to describe the trajectory of social anxiety in postoperative breast cancer patients, explore the influencing factors, and provide theoretical support for the construction of future intervention programs.

**Methods:**

This study was conducted from June 2022 to January 2023, encompassing 213 breast cancer patients from three first-class hospitals in China. Data collection occurred at four distinct time points. A growth mixture model was employed to identify latent categories representing the trajectories of social anxiety changes among patients. A multiple regression analysis was utilized to explore predictive factors associated with different latent trajectory categories.

**Results:**

The trajectory of social anxiety changes in postoperative breast cancer patients includes five potential categories: maintaining mild social anxiety group, changing from mild to moderate social anxiety group, maintaining moderate social anxiety group, changing from moderate to severe social anxiety group, and maintaining severe social anxiety group. Cluster analysis results indicated three types: positive, negative, and low. Logistic regression analysis revealed that younger age, spouses concerned about postoperative appearance, chemotherapy with taxol-based drugs, opting for modified radical surgery or radical mastectomy surgical approaches, and breast cancer patients with negative rumination were factors that influenced patients’ social anxiety (*P* < 0.05).

**Conclusion:**

The trajectory of social anxiety in postoperative breast cancer patients comprises five potential categories. In clinical practice, it is essential to strengthen the management of high-risk populations susceptible to experiencing social anxiety emotions, including younger age, spouses concerned about postoperative appearance, chemotherapy with taxol-based drugs, opting for modified radical surgery or radical mastectomy surgical approaches, and breast cancer patients with negative rumination.

## Introduction

According to the data released by the International Agency for Research on Cancer (IARC) of the World Health Organization (WHO) in 2020, the number of new cases of breast cancer worldwide was as high as 2.26 million, surpassing the incidence rate of lung cancer and becoming the largest cancer type in the world [1]. The typical treatment for breast cancer involves surgery, often complemented by radiation therapy, chemotherapy, and other modalities to enhance patient survival rates. However, these treatments also lead to side effects, such as breast loss, hair loss, nausea, and vomiting, which cause patients to develop social anxiety [2]. Social Anxiety (SA) refers to emotional and behavioral disorders in individuals caused by concerns about negative evaluations from others, leading to a fear of judgment in social situations and a tendency to avoid them actively. If unable to avoid these social situations, individuals may experience physiological reactions such as trembling, stuttering, sweating, blushing, or urgency to urinate. These reactions disrupt interpersonal interactions and affect the individual’s daily life [3]. Studies by Carver et al. [4] suggest that breast cancer patients may experience varying degrees of social anxiety following surgery, potentially associated with altered postoperative body image. If not controlled by timely intervention, social anxiety will not only adversely impact the affected individual but also cause distress to others [5].

Social anxiety is influenced by various factors. Firstly, genetic inheritance is associated with social anxiety. It has been noted that overexpression of genes such as the 5-hydroxytryptamine transporter gene (Solute carrier family 6member4, SLC6A4), catechol-oxygen-site methyltransferase (Catechol-O-methyltransferase, COMT), and Monoamine Oxidase A (MAOA) and other genes may trigger social anxiety in individuals [6]. Secondly, gender also promotes the development of social anxiety. Research on primary school students showed that the level of social anxiety in girls was higher than that in boys [7]. Additionally, social support plays a crucial role in influencing an individual’s level of social anxiety [8]. Rhoten et al. [9] believe that there is a negative correlation between social support and social anxiety, i.e., the more social support, the lower the level of social anxiety. In a cross-sectional study of rumination and social anxiety among college students, researchers concluded that rumination also has an impact on social anxiety and that rumination can affect social anxiety levels among college students directly or indirectly through feelings of loneliness [10]. In an intervention study of rumination and social anxiety among undergraduate psychology students, Mellings [11] revealed that reducing students’ rumination was effective in reducing their focus on negative information about themselves, thereby lowering their levels of social anxiety.

Relevant policies and measures have been implemented to facilitate disease recovery, such as medical insurance coverage for breast cancer patients [12] and postoperative rehabilitation exercise programs [13, 14]. However, specific intervention measures and targeted policies regarding potential social anxiety among patients have not been fully discussed and established. Current research on social anxiety always focuses on healthy populations and is less often applied to diseased groups [15]. Furthermore, existing studies are predominantly cross-sectional studies [16], and previous research has not explored the patterns of changes in social anxiety levels over time and the variations between different populations. Therefore, this study aims to use the Growth Mixture Model (GMM) to investigate potential categories of trajectories in postoperative social anxiety levels among breast cancer patients [17, 18]. Logistic regression will be employed to analyze the influencing factors on the trajectories of social anxiety changes in different potential categories among breast cancer patients. The study will examine the characteristics of high-risk individuals and relevant modifiable factors aiming to facilitate the early identification of high-risk individuals and the implementation of targeted measures. Ultimately, the goal is to reduce social anxiety levels in this high-risk population.

## Methods

### Participants

Breast cancer patients admitted to the surgery wards of three first-class general hospitals in Suzhou from June 2022 to January 2023 were recruited by convenience sampling. The inclusion criteria were: (i) patients diagnosed with primary breast cancer by pathology report who underwent surgical treatment and chemotherapy after surgery; (ii) female, aged 18–75 years old; (iii) with specific communication and comprehension abilities; (iv) who understood their condition and voluntarily participated in this study. The exclusion criteria were: (i) those suffered from malignant tumors in other parts of the body; (ii) combined with other serious diseases (severe heart disease, hypertension, etc.); (iii) withdrew in the middle of the study or interrupted the treatment for various reasons; (iv) receiving any psychological therapy or counseling intervention during treatment; (v) with a history of mental illness before surgery.

The study was ethically approved by the Ethics Committee of Soochow University (SUDA20230115H05). All patients completed four time-point measurements: the day of admission (T1), seven days postoperative (T2), one month postoperative (T3), and three months postoperative (T4). This study employed a longitudinal research design, estimating the sample size based on the repeated measures design proposed by Barcikovski and Robey [19]. The sample size was calculated using GPower software, with an effect size of 0.1 [20–22], a significance level (α) of 0.05, a power (1-β) of 0.95, and a calculated sample size of 431 participants. Because the number of repeated measurements was 4, the number of study subjects was calculated to be 108. Because this study involves fitting a GMM. Research indicates that when using the Bayesian Information Criterion as the primary consideration for model selection, the sample size should be ≥ 200 [21, 23]. Therefore, the sample size for this study should be at least 200 cases.

### Study measures

#### General information questionnaire

Two components were included: socio-demographic information and disease information. Socio-demographic data included age, ethnicity, whether there was religious belief, occupation, work status, marital status, education level, per capita monthly family income, place of residence, whether there were children, and whether the spouse cared about the patient’s image after surgery. Disease and treatment information included the affected side, family history, tumor stage, metastasis, surgical method, chemotherapy medication, and whether other diseases were combined.

#### Positive and negative rumination scale

It included 23 entries, which were categorized into five dimensions: enjoyment of pleasure, inhibition of pleasure, denial of self, positive coping, and negative attribution [24]. Enjoyment of pleasure and positive coping constitute the positive rumination degree, whereas the remaining three factors constitute the negative rumination degree. The scale is scored using a four-point scoring system, with options A-D corresponding to scores of 1–4, respectively. The total sum of these scores represents the final score for each dimension [24]. The internal consistency coefficients of the scale ranged from 0.70 to 0.81, with the internal consistency coefficients for negative and positive rumination being 0.84 and 0.81 and the internal consistency coefficient for the total scale amounting to 0.74 [25, 26]. For distinguishing between types of rumination, we followed the approach outlined by Yang et al. [27]. The specific steps are as follows: (1) Calculate the standard scores, or Z-scores, for the two dimensions of positive and negative rumination; (2) Utilize cluster analysis to determine the number of clusters by assessing the maximum change in the agglomeration coefficient between clusters, using the sum of squared deviations and Euclidean distance. Subsequently, individuals are grouped into several categories based on these clusters.

#### Interaction anxiety scale

It is mainly used to measure individuals’ perceived social anxiety levels in interpersonal interactions but does not measure specific external social behaviors [28]. The scale consists of 15 self-report items, with options A-E corresponding to scores of 1–5, respectively. The total sum of these scores represents the final score, which ranges from 15 to 75 points. The scale has high reliability and validity, with Cronbach’s alpha coefficient greater than 0.87 and an 8-week retest correlation coefficient of 0.80. A score of less than or equal to 31 is classified as a low social anxiety group, a score of 32–48 is classified as a medium anxiety group, and a score of more than 48 is classified as a high social anxiety group.

### Data analysis

The latent classes of social anxiety trajectories were identified using GMM. The fitting process began with a one-class latent model (1 C), during which relevant fit indices were summarized to determine the most suitable fitting model. Considering group heterogeneity, identifying potential types was followed by exploring the characteristics of each group of patients [29]. A Robust Maximum Likelihood Estimator (MLR) is used to estimate parameters and handle missing values in the follow-up data, assuming the randomness of missing values did not affect the results. When fitting the GMM model, the metrics for evaluating the goodness-of-fit include (i) loglikelihood value (Loglikelihood, LL), AIC (Akaike Information Criterion), and sample size-adjusted BIC (sample size-adjusted BIC) information indices. These statistics are used to judge the goodness of fit by comparing the expected and actual values, and the smaller the value, the better the fit. (ii) Classification accuracy can be evaluated by entropy, which ranges from 0 to 1. Entropy ≥ 0.80 indicates that the classification accuracy is more than 90%, and the closer the entropy value is to 1, the more accurate the classification is. (iii) In addition, Bootstrapped Likelihood Ratio Test (BLRT) and Vuong-Lo-Mendell-Rubin Likelihood Ratio Test (VLMR) are used to compare the number of different categories of the models. These tests determine whether the difference in fit between a K-category model and a K-1-category model is significant. If the *p*-values of the BLRT and VLMR tests are insignificant, the K-category model is not better than the K-1-category model [30]. Cluster analysis was employed to categorize and summarize the types of rumination in breast cancer patients. Normally distributed data were expressed as mean and standard deviation, and multiple comparisons were made using ANOVA. Count data were presented as frequencies and percentages, and chi-square tests were used for multiple group comparisons. Finally, regression analysis was used to explore the effects of socio-demographic, disease-related information, and different types of rumination on the latent categories trajectories of social anxiety changes. Differences were considered statistically significant at *P* < 0.05. Mplus 7.4 and SPSS 25.0 were used for data analysis.

## Results

**Fig. 1:**
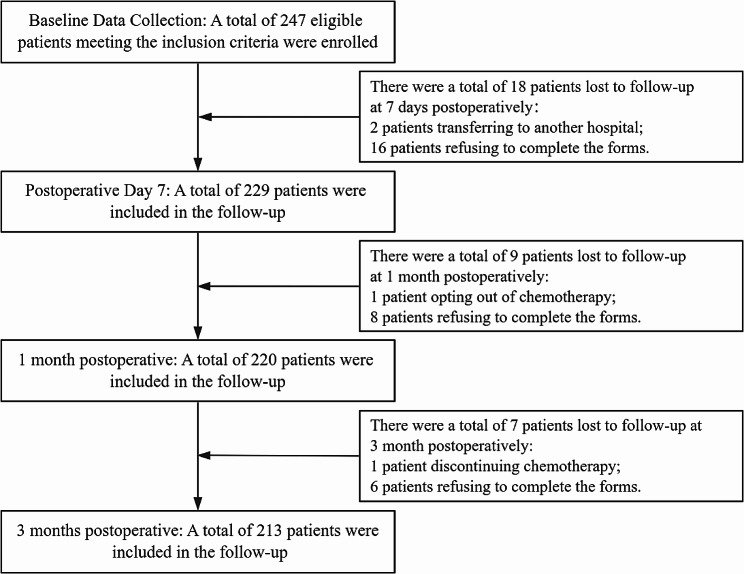
The enrollment and attrition of patients during the study process

In the baseline data survey, a total of 247 patients were enrolled, with 213 ultimately completing all four surveys. During the follow-up process, there were patients lost to follow-up. Reasons for loss to follow-up included patients discontinuing treatment, refusing to fill out forms, and seeking treatment at another hospital. The follow-up process is illustrated in Fig. 1. The results revealed that the participants had an average age of 47.9 ± 9.70 years old, the youngest being 24 years old and the oldest being 72 years old. (Table 1)

**Table 1:** Basic characteristics of the included participants

Factors	Inclusion of 213 Patients	
	n/Mean	%/SD
Age	48.02	9.77
Occupations		
Staff	87	40.8%
Worker	46	21.6%
Farmer	17	8.0%
Self-Employed Entrepreneur	22	10.3%
Others	41	19.2%
Employment Status		
Employed	115	54.0%
Retired	75	35.2%
Unemployed	15	7.0%
Never Employed	8	3.8%
Marital Status		
Single	8	3.8%
Married	198	93%
Divorced	7	3.3%
Widowed	0	0%
Education Level		
Elementary School	29	13.6%
Junior High School	79	37.1%
Senior High School / Vocational School	50	23.5%
University	50	23.5%
Graduate and Above	5	2.3%
Menstrual		
Non-menopausal, regular	92	43.2%
Non-menopausal, irregular	31	14.6%
Natural menopause	86	40.4%
Hysterectomy	4	1.9%
The spouse cares about the current image		
Yes	93	43.7%
No	118	56.3%
Affected Side		
Left	105	49.3%
Right	105	49.3%
Bilateral	3	1.4%
Comorbidity		
Yes	34	16%
No	179	84%
Tumor Staging		
Stage I	36	16.9%
Stage II	140	65.7%
Stage III	37	17.4%
Stage IV	0	0%
Metastasis		
Yes	32	15%
No	181	85%
Family History		
Yes	52	24.4%
No	161	75.6%
Chemotherapeutic regimen		
Taxol	135	63.4%
Anthracycline	78	36.6%
Surgical Procedures		
Modified radical surgery	116	54.5%
Breast-conserving surgery	59	27.7%
Radical resection	38	17.8%

### Potential categories of change trajectories in social anxiety in postoperative breast cancer patients

**Fig. 2:**
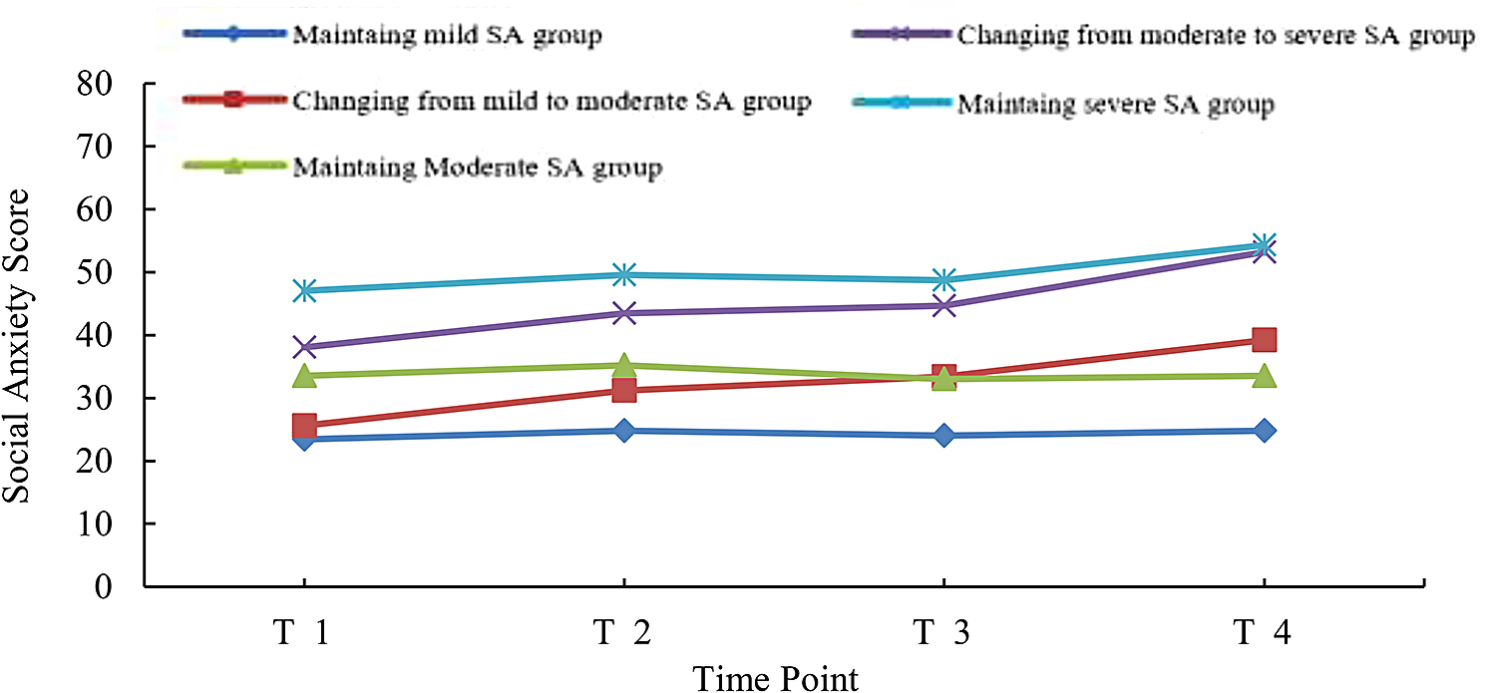
Distribution of GMM five categories of social anxiety levels in postoperative breast cancer patients Note: T1: The day of admission; T2: Seven days postoperative; T3: One month postoperative; T4: Three months postoperative

A total of six potential category models were fitted in this study, and the relevant fit indices are in Table 2. The results showed the best fit to the data for five potential category models, each named according to the characteristics of the trajectory of change in social anxiety, namely Marinating mild SA group (37.95), Changing from mild to moderate SA group (7.2%), Maintaining moderate SA group (31%), Changing from moderate to severe SA group (15.2%), and Maintaining severe SA group (8.8%). (Fig. 2).

**Table 2:** Fitting results of latent class models of social anxiety trajectories in postoperative breast cancer patients

GMM	LL	AIC	BIC	ABIC	entropy	BLRT *p*	VLMR *p*
1 C	-2640.97	5305.93	5347.09	5309.05			
2 C	-2614.31	5258.62	5310.06	5262.52	0.84	<0.001	<0.001
3 C	-2603.84	5243.69	5305.42	5248.37	0.75	<0.001	<0.001
4 C	-2595.59	5233.19	5305.20	5238.65	0.80	<0.001	<0.001
5 C	-2583.31	5214.63	5296.93	5220.87	0.85	<0.001	<0.001
6 C	-2580.87	5215.73	5308.33	5308.33	0.82	1.000	<0.001

Note: Growth Mixture Model (GMM); Log Likelihood Function (LL); Akaike Information Criterion (AIC); Bayesian Information Criterion (BIC); Bootstrapped Likelihood Ratio Test (BLRT); Likelihood Ratio Test (Vuong-Lo-Mendell-Rubin, VLMR). By comparing the values of AIC, BIC, ABIC, entropy, BLRTp, and VLMRp in the different models, it was finally determined that the data of the five potential category models fitted the best. This is because the five potential category models had the smallest values of BIC and ABIC, the largest values of entropy and met the criteria of entropy ≥ 0.8, and BLRTp and VLMRp were less than 0.05 categorically significant. Although the entropy values of two, four, and six potential category models were all greater than 0.8, the values of AIC, BIC, and ABIC in these groups were larger than those of the five potential category models. The VLMRp of five potential category models is greater than 0.05, and only five potential category models satisfy the condition

### Rumination type in breast cancer patients

**Fig. 3:**
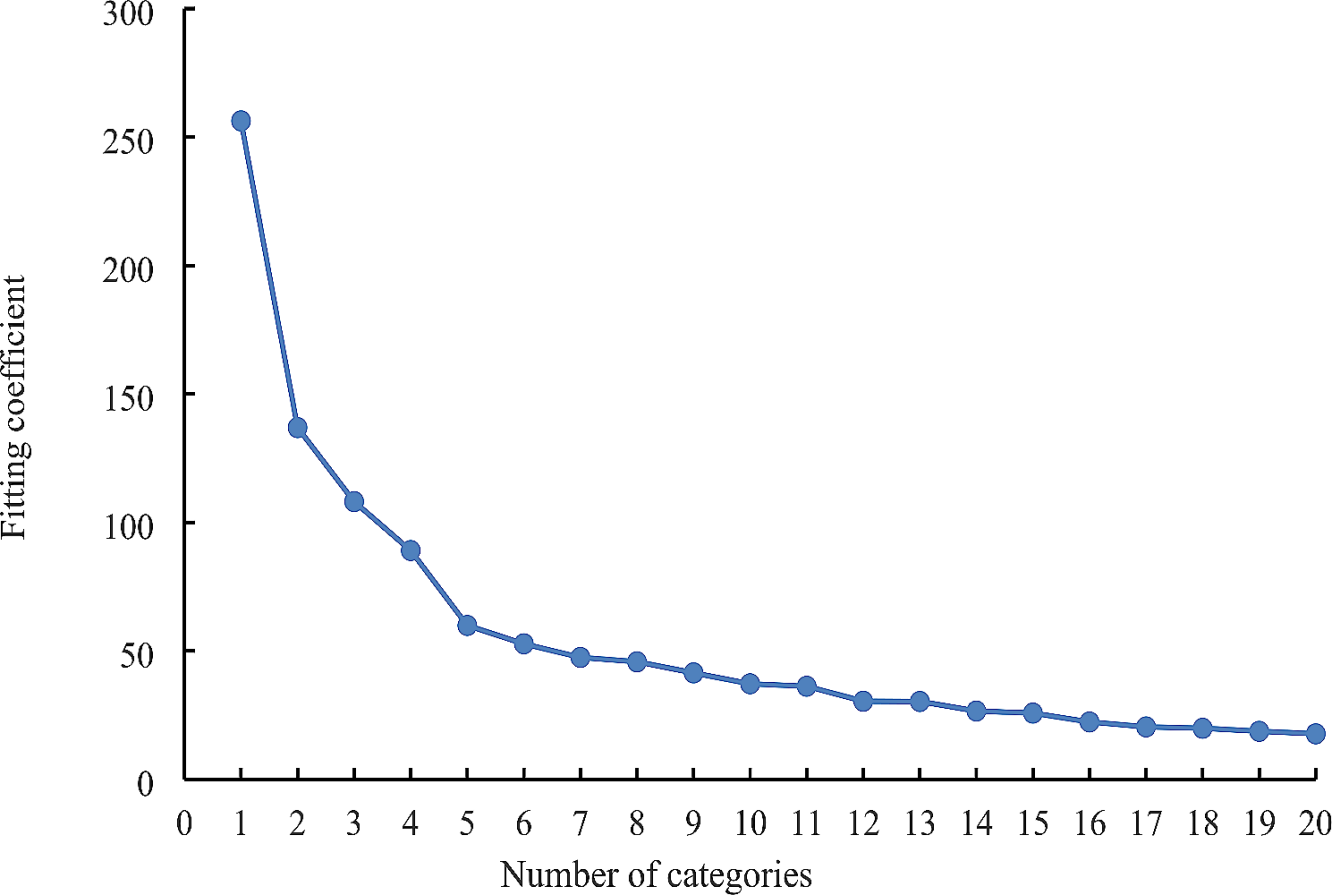
Cluster analysis of the variation of agglutination index fold plot Note: Determine the number of categories for rumination. The results showed that the movement of the agglutination index changed most steeply between 2 and 3, so the number of clusters should be between 2 and 3, and synthesizing previous studies, the number of categories was finally set to 3

**Fig. 4:**
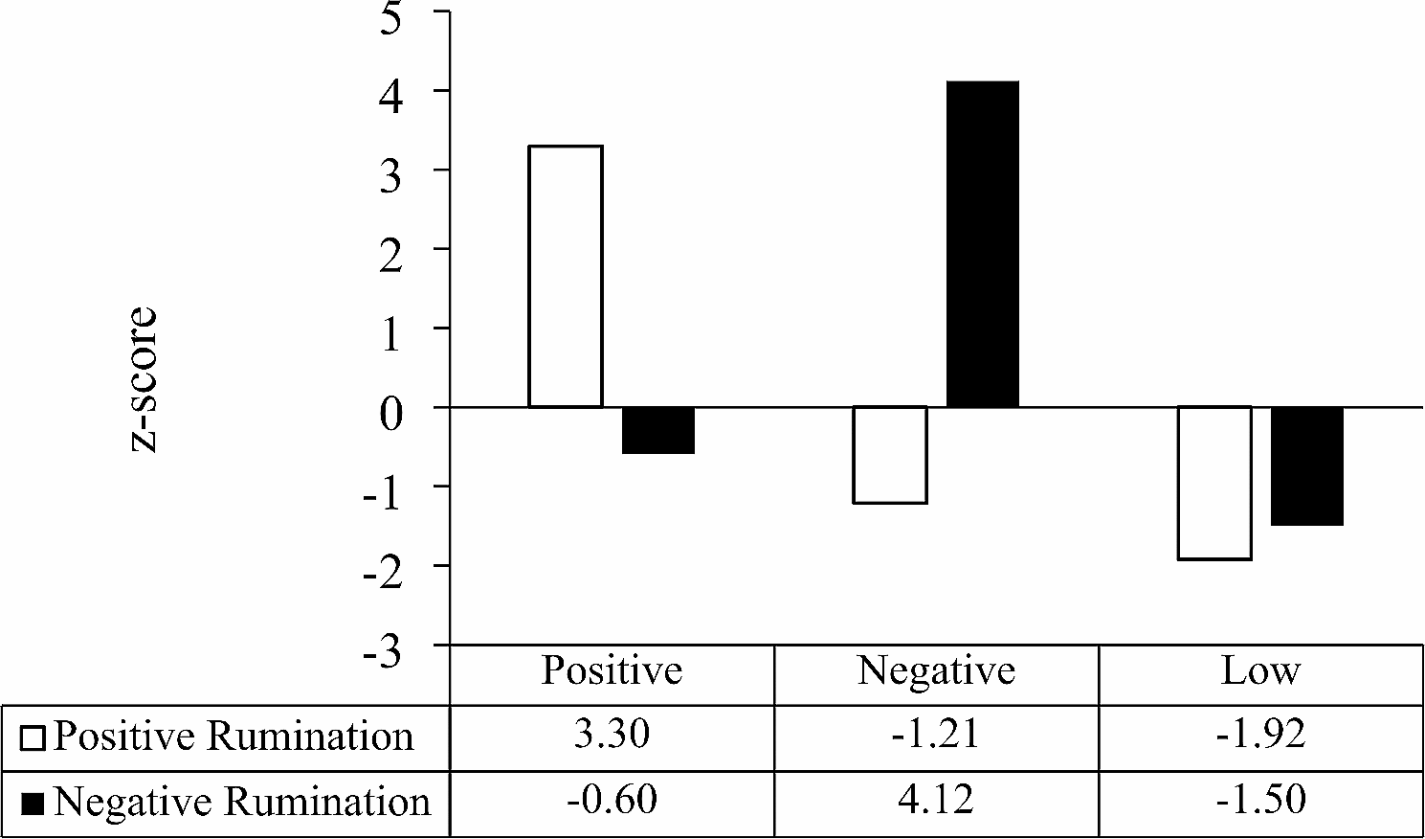
Final clustering of the three types of rumination Note: Distinguish between three types of ruminators. The standardized scores (Z-scores) of positive and negative rumination scores were clustered using K-means clustering and clustered into three categories, positive, negative and low. The table shows that the positive ruminator has positive values for their positive rumination and negative values for their negative; on the contrary, the negative ruminator has positive values for their negative rumination and negative values for their positive rumination; whereas the low type indicates that this category is in a neutral state so that they have negative values for both positive and negative aspects

Since the total score of the scale used in this study contains both positive and negative aspects and therefore has less significance for its mean value, this study analyzed the positive and negative rumination of 213 patients in a hierarchical cluster analysis to categorize the level of rumination of the included population. The results showed that among the 213 breast cancer patients who completed all the follow-ups, 32.4% of the patients had a positive type, 32.4% had a negative type, and the remaining 35.2% had low type of rumination. (Figures 3 and 4).

### Univariate analysis of factors influencing potential categories of social anxiety change trajectories in postoperative breast cancer patients

The results of the univariate analysis showed that menstrual status, whether the spouse cares about the postoperative image, chemotherapy regimen, surgical approach, and the type of rumination affect the potential categories of social anxiety change trajectories in postoperative breast cancer patients. (Table 3).

**Table 3:** Results of univariate analysis of factors affecting social anxiety in postoperative breast cancer patients

Factors	Maintaining mild SA group (*N* = 15)	Changing from mild to moderate SA group (*N* = 71)	Maintaining moderate SA group (*N* = 16)	Changing from moderate to severe SA group (*N* = 79)	Maintaining severe SA group (*N* = 32)	*p*
Menstrual						<0.001
Non-menopausal, regular	21(26.6%)	10(66.7%)	35(49.3%)	18(56.3%)	8(50%)	
Non-menopausal, irregular	7(8.9%)	1(6.7%)	13(18.3%)	7(21.9%)	3(18.8%)	
Natural menopause	50(63.3%)	4(26.7%)	22(31%)	5(15.6%)	5(31.3%)	
Hysterectomy	1(1.3%)	0(0%)	1(1.4%)	2(6.3%)	0(0%)	
The spouse cares about the current image						<0.001
Yes	12(15.2%)	9(60%)	34(47.9%)	25(78.1%)	13(81.3%)	
No	67(84.8%)	6(40%)	37(52.1%)	7(21.9%)	3(18.8%)	
Chemotherapeutic regimen						0.001
Taxol	42(53.2%)	15(100%)	41(57.7%)	22(68.8%)	15(93.8%)	
Anthracycline	37(46.8%)	0(0%)	30(42.3%)	10(31.3%)	1(6.3%)	
Surgical Procedures						<0.001
Modified radical surgery	34(43%)	12(80%)	41(57.7%)	23(71.9%)	6(37.5%)	
Breast-conserving surgery	33(41.8%)	1(6.7%)	22(31%)	3(9.4%)	0(0%)	
Radical resection	12(15.2%)	2(13.3%)	8(11.3%)	6(18.8%)	10(62.5%)	
Types of rumination						<0.001
Positive	24(30.4%)	1(6.7%)	26(36.6%)	0(0%)	3(18.8%)	
Negative	6(7.6%)	13(86.7%)	21(29.6%)	29(90.6%)	10(62.5%)	
Low	49(62.0%)	1(6.7%)	24(33.8%)	3(9.4%)	3(18.8%)	

### Logistic regression analysis of factors influencing social anxiety in postoperative breast cancer patients

Statistically significant independent variables identified through univariate analysis included menstrual status, whether the spouse cared about the postoperative image, chemotherapy regimen, surgical approach, and type of rumination. These metrics were subjected to logistic regression analysis to determine the final influences affecting the potential categories of social anxiety change trajectories in postoperative breast cancer patients. The results showed that the younger the age, the more likely their social anxiety trajectory would develop into the changing from moderate to severe SA group; patients whose spouses cared about their postoperative image would develop into the changing from mild to moderate SA group, maintaining moderate SA group, changing from moderate to severe SA group, and maintaining severe SA group; patients whose chemotherapeutic agents contained paclitaxel were more likely to develop a trajectory of social anxiety into changing from moderate to severe SA group and maintaining severe SA group; while patients with breast-conserving surgery were more likely to be in the maintaining mild SA group; breast cancer patients with negative ruminators were more likely to develop changing from mild to moderate SA group, changing from moderate to severe SA group and maintaining severe SA group. (Table 4).

**Table 4:** Logistic regression analysis of factors influencing social anxiety change in postoperative breast cancer patients

Latent class	Affect variables		B	WALD	OR	95% Confidence Interval	*p*
Maintaining mild SA group	Negative rumination		-1.709	10.044	0.181	0.06–0.52	0.002
Changing from mild to moderate SA group	Whether the spouse cares about the current image	Yes	1.64	4.89	5.15	1.21–22.03	0.027
	Negative rumination		2.71	6.23	15.09	1.79–127.14	0.010
Maintaining moderate SA group	Whether the spouse cares about the current image	Yes	1.05	5.28	2.84	1.17–6.93	0.022
Changing from moderate to severe SA group	Whether the spouse cares about the current image	Yes	2.31	13.20	10.09	2.90–35.09	<0.001
	Surgical approach	Breast-conserving surgery	-2.34	6.54	0.10	0.02–0.58	0.007
	Chemotherapy regimens	Taxol	1.122	4.019	3.072	1.03–9.21	0.045
	Age		-0.112	5.989	0.894	0.82–0.98	0.014
	Negative rumination		2.249	10.829	9.476	2.49–36.17	0.001
Maintaining severe SA group	Whether the spouse cares about the current image	Yes	3.18	13.19	23.98	4.32–133.25	<0.001
	Chemotherapy regimens	Taxol	2.54	4.95	12.73	1.35–119.56	0.026
	Negative rumination		1.43	3.77	4.17	0.99–17.60	0.050

## Discussion

This study conducted four follow-up surveys on the level of social anxiety among breast cancer patients three months post-surgery. Results indicated that over half of the postoperative breast cancer patients experienced moderate to severe levels of social anxiety, and the trajectory of social anxiety comprises five potential categories. Additionally, this study revealed that younger age, spouse’s concern about the patient’s postoperative appearance, chemotherapy regimen containing taxane drugs, surgical methods other than breast-conserving surgery, and breast cancer patients with negative rumination tendencies were highly prone to experiencing social anxiety.

This study found the best models after potential category analysis, including maintaining mild SA group (37.95%), changing from mild to moderate SA group (7.2%), maintaining moderate SA group (31%), changing from moderate to severe SA group (15.2%), and maintaining severe SA group (8.8%). The maintaining mild SA group and maintaining moderate SA group demonstrated relatively stable and minor changes in social anxiety levels throughout the entire treatment process. Which may have increased slightly postoperatively but remained below the threshold for moderate or severe social anxiety consistently maintaining a low to remaining moderate social anxiety, similar to the baseline level. The changing from mild to moderate SA group and the changing from moderate to severe SA group exhibited consistently mild to moderate levels of social anxiety before surgery. Due to the psychological impact of the surgery, these patients experienced a significant increase in postoperative social anxiety levels. In some cases, individuals in the moderate social anxiety group reached levels indicative of severe social anxiety. Patients with severe social anxiety, whether preoperative or postoperative, consistently exhibited relatively high levels of social anxiety. The study has confirmed that people with severe social anxiety are susceptible to adverse evaluations. When confronted with negative emotions or evaluations from the external environment, they rapidly perceive and generate anxiety emotions [31]. In this study, 62% of the patients experienced moderate to high levels of social anxiety, aligning closely with previous research findings [32].

Age, the spouse’s care about the postoperative image, chemotherapy regimen, surgical modality, and type of rumination are all influential factors in the change of social anxiety in postoperative breast cancer patients. First, younger patients tend to have social anxiety trajectories that are more likely to develop into changing from moderate to severe SA group. Nowadays, the expectations for women are increasing, requiring them to be not only intelligent but also attentive to their appearance. Younger women are becoming increasingly demanding and paying more and more attention to their appearance. The current treatment methods for breast cancer can cause substantial harm to the external appearance of younger women, leading to a sense of shame, which is highly likely to result in social anxiety [33, 34]. On the contrary, older individuals have a more negligible probability of developing such negative emotions [35]. This may be because older people can approach illness more rationally. Therefore, compared to younger women, their changes in social anxiety levels are less noticeable. Research has also confirmed that younger individuals are more prone to social anxiety and sensitive to social relationships [36]. Despite the favorable prognosis of disease after treatment for younger breast cancer patients, the damage to their appearance caused by the treatment is also very vulnerable to negative evaluation by others, and such negative evaluation leads to an increase in the level of social anxiety in young postoperative breast cancer patients.

Second, patients who believed that their spouses were concerned about their current appearance tend to experience an increase in social anxiety levels as the disease progresses. The results of this study are like those of Li Jianghua [32]. Such patients who care about their spouse’s perception of their image are more likely to develop social anxiety after breast cancer surgery. Breast cancer is a stressful event experienced by both spouses, and spousal support can help patients reduce psychological pressure [37, 38]. Since breast cancer patients are predominantly female, after experiencing postoperative appearance changes, patients will have a psychological burden. Without timely and positive guidance from their spouses, patients may engage in negative behaviors such as avoidance of communication [39]. Some studies have shown that adequate social support can reduce the level of social anxiety [40], with family support, particularly spousal support, playing a crucial role in coping with social anxiety emotions [41]. A qualitative study on breast cancer patients post-surgery suggested that some patients believed their spouses were disgusted with their appearance, leading to feelings of self-loathing and guilt [42]. Patients’ spouses indicated that the patient was less attractive after surgery, which led to the gradual dissolution of the intimate relationship between the spouses in a vicious circle [43]. The lack of social support makes patients more prone to negative emotions and inferiority [44], which makes them reluctant to contact the outside world and close themselves off, leading to social withdrawal and heightened levels of social anxiety [45].

Patients receiving taxane chemotherapy, such as paclitaxel, are more likely to develop change from the moderate to severe SA group and maintain the severe SA group. This may result from the mechanism of action of taxane drugs on cancer cells. Taxane drugs, including paclitaxel and docetaxel, primarily function by inhibiting the mitosis of tumor cells, thereby suppressing their proliferation [46]. The main adverse reactions include neurotoxicity, myelosuppression, and allergic reactions. Notably, neurotoxicity is the most prominent, manifesting as peripheral neuropathy, sensory neuropathy, and other symptoms [47], which can affect patients’ quality of life and social function in severe cases [48]. Taxane drugs are neurotoxic, causing neurological-related discomfort and producing chronic neurotoxic clinical symptoms, such as unrelenting pain sensation, weakness, and fatigue. This affects patients’ moods and psychological states, leading to negative emotional states such as fear of socialization and depression [49, 50].

The surgical procedure is a factor Influencing social anxiety among breast cancer patients. Breast cancer patients treated with breast-conserving surgery were more likely to develop into the maintaining mild SA group. The loss of the breast caused by breast cancer surgery is a significant factor influencing patients’ development of social anxiety. A Greek research study suggested that patients undergoing mastectomy and breast-conserving surgery would experience different emotions. Patients undergoing radical mastectomy were more likely to believe they were unattractive, had increased self-consciousness, were dissatisfied with their appearance, and avoided contact with others compared to those treated with breast-conserving therapy [51]. Although modified radical mastectomy can achieve radical tumor cure or significantly prolong patient survival time and protect the patient’s physiological function, this procedure cannot maintain the integrity of the breast, leading to negative emotions due to physical damage [52]. Isabel suggested that patients who undergo mastectomy would experience adverse effects on body perception and social behavior, severely affecting the patient’s quality of life [53]. Compared to other procedures, only breast-conserving surgery preserves breast morphology, helping patients alleviate postoperative psychological stress and maintaining social anxiety levels postoperatively. Shi et al. [54] confirmed this viewpoint in a two-year follow-up study involving 172 breast cancer patients. The result showed that patients undergoing breast reconstruction had the highest quality of life scores, followed by patients undergoing breast-conserving surgery, while patients choosing mastectomy had the lowest quality of life scores. Therefore, Shi et al. [54] concluded that patients undergoing mastectomy for breast cancer would have adverse impacts on their quality of life, leading to an increase in their levels of social anxiety [54, 55].

Finally, an individual’s rumination type also affected the social anxiety of the patients. The results showed that negative ruminators showed a gradual increase in their postoperative social anxiety levels, while positive and low ruminators tended to have a more stable trajectory of social anxiety emotions. This may be due to the stability of the memory-refreshing function of such people after being harmed by adverse events or negative emotions in positive and low types, which helps them control the production of negative emotions [56]. This is consistent with the findings of Yang [27], who concluded that positive ruminators are predictive of positive psychological indicators and that positive types react quickly when they encounter adverse events to regulate possible negative emotions. In contrast, patients with negative rumination showed an increase in postoperative social anxiety, which is consistent with the results of Jolijn [57] and Faith A [58]. Who found that people with high negative rumination also had relatively high social anxiety. Compared to the positive type, negative ruminators will be stuck in a negative emotion that cannot be extricated, and they will keep thinking about every detail after each socialization, which will produce a negative evaluation of themselves and increase the individual’s level of social anxiety [59]. Research suggests that negative ruminators have weaker emotional regulation abilities, are more susceptible to adverse events, and generate negative emotions [60].

### Limitations

This study was conducted only in first-class hospitals in Suzhou due to epidemic and time factors. In the future, we may further consider expanding the scope of the study, conducting a multicenter, large-sample study, increasing the follow-up time, and including postoperative breast cancer patients with six or eight cycles of chemotherapy to validate the results of this study. Based on the results of this study, a social anxiety management program for postoperative breast cancer patients can be constructed and evaluated for clinical practice.

## Conclusions

There are dynamic changes in the level of social anxiety in postoperative breast cancer patients with different potential categories. In clinical practice, we should pay more attention to breast cancer patients who are younger, whose spouses are concerned about the patient’s image, whose chemotherapy regimens contain paclitaxel, whose surgical methods, and who have negative rumination. To help the clinic identify the characteristics of the postoperative population that is prone to social anxiety at an early stage and to provide references for the development of corresponding nursing measures.

## Data Availability

The authors have full control of all primary data available from the corresponding author (tianlisz@suda.edu.cn) upon request.

## Abbreviations

SA: Social Anxiety
GMM: Growth Mixture Model
BLRT: Bootstrapped Likelihood Ratio Test
VLMR: Vuong-Lo-Mendell-Rubin Likelihood Ratio Test
